# From the archives: Boosting rice immunity, phosophoglucose isomerases influencing seed yield, and regulation of mRNA splicing

**DOI:** 10.1093/plcell/koad174

**Published:** 2023-06-22

**Authors:** Nicolas M Doll

**Affiliations:** Assistant Features Editor, The Plant Cell, American Society of Plant Biologists, Rockville, MD, USA; Department of Plant Biotechnology and Bioinformatics, Ghent University, Ghent 9052, Belgium; VIB Center of Plant Systems Biology, Ghent 9052, Belgium

## September 2022: Boosting rice immunity to fight the blast fungus

**Figure 1.**
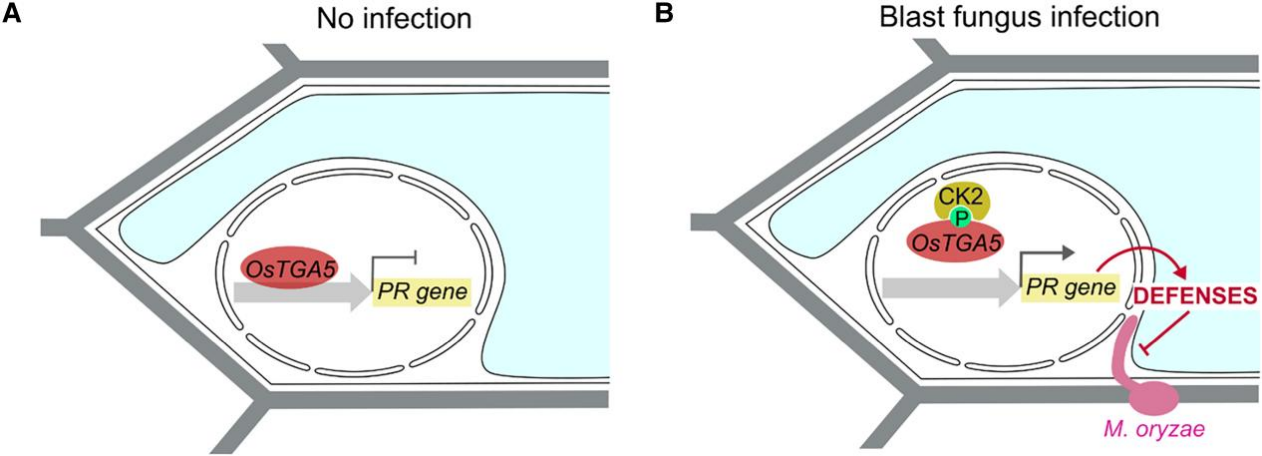
The OsTGA5/CK2 module regulates rice defenses against *M. oryzae*. **A)** In the absence of blast fungus infection, OsTGA5 inhibits the expression of PR genes through direct binding of their promoters. **B)** Upon infection by the blast fungus, CK2 phosphorylates OsTGA5, reducing its binding affinity to the PR promoters, which releases the transcriptional repression of PR genes and activates the defense response. Created with Inkscape by N. M. Doll.

Rice blast disease caused by the ascomycete *Magnaporthe oryzae* represents a worldwide threat to rice culture. To fight it, the plant sets up immune responses that involve the expression of pathogenesis-related (PR) genes. Boosting these responses represents a way to generate blast-resistant cultivars. **Yuqing Niu and colleagues (Niu et al. 2022)** showed that the transcription factor OsTGA5 acts as a negative regulator of rice defenses against the blast fungus. *Ostga5* mutant plants displayed increased resistance to *M. oryzae* and higher expression levels of many PR genes. In healthy plants, OsTGA5 binds the promoter of *JIOsPR10*, a key gene for rice immunity (Wu et al 2016), and represses its expression. During blast fungus infection, this repression is released. The authors showed by genetic and biochemical approaches that *M. oryzae* infection triggers the phosphorylation of OsTGA5 by CASEIN KINASE II (CK2), which reduces its binding affinity for the *JIOsPR10* promoter, thereby releasing the repression of *JIOsPR10* and other *PR* genes (Fig. 1). Similar to the *Ostga5* mutant, *CK2* overexpression in rice boosted the defense against *M. oryzae*. In summary, manipulation of the CK2/OsTGA5 module appears to be an efficient way to generate blast-resistant rice plants. Identifying the potential trade-off caused by this manipulation and testing the consequences on the plant defenses against other pathogens will be needed to assess the potential for generating blast-resistant rice cultivars.

## September 2018: Plastidial PGI1 regulates seed yield through numerous metabolic pathways

Seed yield is determined by both the number of seeds produced by a plant and the quantity and quality of the nutrients stored. In this article, **Abdellatif Bahaji and colleagues (Bahaji et al. 2018)** determined the contribution of PHOSPHOGLUCOSE ISOMERASE ISOFORM 1 (PGI1) to seed yield in Arabidopsis. PGI1 catalyzes the isomerization of glucose-6P into fructose-6P, a critical step in numerous metabolic pathways, including the glycolysis/oxidative pentose phosphate pathway, and the biosynthesis of fatty acids, proteins, and isoprenoid hormones like cytokinins and gibberellins (GA). The Arabidopsis genome encodes 2 PGI proteins: cytoplasm-localized PGI and plastidial PGI1. The seed yield of the *pgi1* mutants was strongly reduced for different reasons. First, *pgi1* mutant plants produce fewer inflorescences and siliques per inflorescence, leading to fewer seeds per plant. In the *pgi1* inflorescence, bioactive GA levels are reduced and the application of exogenous GA reverts the phenotype, indicating that impaired GA biosynthesis causes this trait. Second, *pgi1* mutants display lower levels of fatty acids and storage proteins in the embryo, interpreted as being caused by the limited entry of carbon atoms into the glycolysis/oxidative pentose phosphate pathway/Rubisco network. In conclusion, PGI1 contributes to different aspects of seed yield through diverse metabolic pathways in plastids. Interestingly, mutation of the cytoplasmic PGI in Arabidopsis is lethal during microsporogenesis and embryogenesis, indicating an even more drastic contribution of the cytoplasmic PGI to plant reproduction (Liu et al 2023). These 2 studies offer a nice overview of PGI functions in plant reproduction and seed yield.

## September 1998: Intronic sequences are important for mRNA splicing in plants—the case of AP3

Correct splicing of precursor messenger RNAs (mRNAs) is essential for the production of mature mRNAs by the plant cell. In this article, **Ying Yi and Thomas Jack (Yi and Jack 1998)** analyzed the splicing of *APETALA 3* (*AP3*), a gene involved in flower morphogenesis in Arabidopsis. In an *ap3-1* mutant, a point mutation located 2 bases before the 3’ end of the fifth exon alters the consensus sequence for splicing. Consequently, many *AP3-1* mRNAs in the mutant do not have a fifth exon, which results in decreased levels of functional AP3 proteins in flowers, causing an intermediate phenotype between wild-type and full knock-out mutants (Sablowski and Meyerowitz 1998). To find potential mutations that would suppress the *ap3-1* mutant phenotype, the authors carried out EMS mutagenesis in *ap3-1*. They identified 4 independent suppressor mutants that all bear the same intragenic mutation, namely a single nucleotide substitution in intron 4, 33 bases upstream of exon 5. This mutation significantly increases the proportion of *AP3-1* mRNAs with a correctly spliced fifth exon. The authors suggested that this mutation creates a novel branch point sequence in intron 4 that enhances exon 5 splicing. This result demonstrated the importance of intronic sequences in the regulation of mRNA splicing in plant cells.
